# Topical, contact, and oral susceptibility of adult *Culicoides* biting midges (Diptera: Ceratopogonidae) to fluralaner

**DOI:** 10.1186/s13071-023-05899-7

**Published:** 2023-08-14

**Authors:** Blythe E. Lawson, Emily G. McDermott

**Affiliations:** Department of Entomology and Plant Pathology, University of Arkansas, Fayetteville, AR 72701 USA

**Keywords:** Isoxazoline, Vector control, Ceratopogonidae, Integrated pest management

## Abstract

**Background:**

*Culicoides* biting midges (Diptera: Ceratopogonidae) are economically important blood-feeding pests closely associated with livestock production. They are the principal vectors of two hemorrhagic disease viruses affecting both wild and domestic ruminants within the US: bluetongue virus (BTV) and epizootic hemorrhagic disease virus (EHDV). BTV impacts the US agriculture sector through direct commodity loss and strict international livestock trade restrictions. Yet, despite posing a considerable threat to US livestock, *Culicoides* are understudied, and management strategies are lacking. Current control tools for *Culicoides* are limited to synthetic chemicals, predominantly pyrethroids. With limited products available for livestock producers, proper pesticide rotation is difficult. The present study investigates the efficacy of fluralaner, an isoxazoline insecticide, beyond its current labeled use as an ectoparasiticide in anticipation of adding a new class of pesticides into rotation for use against biting midges.

**Methods:**

The efficacy of fluralaner was evaluated by conducting contact, topical, and oral toxicity bioassays on adult female *Culicoides sonorensis*. Contact toxicity was assessed by using a modified WHO cone assay, which simulates exposure through landing on an insecticide-treated surface. A modified WHO topical toxicity assay, in which fluralaner dilutions were administered to the lateral thorax, was used to assess topical toxicity. For evaluation of oral toxicity, females were offered a blood meal spiked with fluralaner in an artificial membrane feeding system to simulate a systemic insecticide.

**Results:**

Contact exposure of fluralaner did not cause extensive or consistent mortality. Even the highest concentration tested (100 mg/ml) resulted in an average of only 24.3% mortality at 24 h, and mortality did not significantly differ between exposed and control midges at any concentration. One hundred percent mortality was consistently achieved at concentrations of 1 mg/ml when fluralaner was applied topically. The LC_50_ for topical exposure to fluralaner at 24 h was estimated to be 0.011 mg/ml. Oral exposure to fluralaner through ingestion of a spiked blood meal proved to be the most effective exposure method, significantly increasing mortality in a dose-dependent manner at 1 h post-exposure. The LC_50_ at 24 h following ingestion was 14.42 ng/ml.

**Conclusion:**

Our results suggest that fluralaner is a viable candidate for use as an insecticide against adult biting midges if exposed orally, such as in a systemic given to livestock. As withdrawal period requirements for meat animals present unique yet definitive challenges, pharmacokinetic studies of isoxazoline drugs need to be pursued and finalized for livestock before fluralaner may be used as a management strategy in this manner. Alternatively, livestock not raised for consumption, such as hair sheep, would directly benefit from administering oral fluralaner as a component of a BTV disease management program.

**Graphical abstract:**

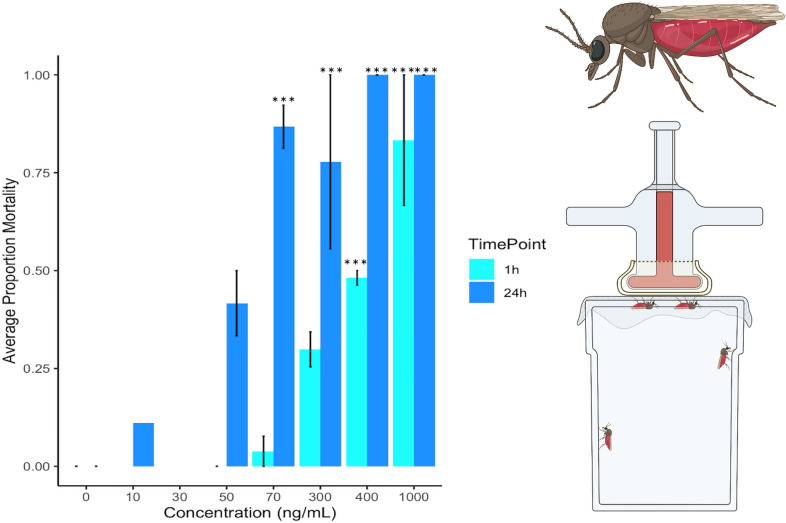

## Background

*Culicoides* biting midges (Diptera: Ceratopogonidae) are minute biting flies that transmit bluetongue virus (BTV) to domestic and wild ruminants [1–3]. This viral pathogen belongs to the family *Reoviridae* and causes symptoms characteristic of hemorrhagic viral diseases such as fever, lameness, internal hemorrhage and death in the animal host [4–6]. Of the susceptible domestic ruminants, sheep suffer the highest morbidity and mortality. Cattle are typically asymptomatic, but infections in pregnant cows can result in fetal abnormalities and abortion, and subclinical infections can reduce milk yields and feed conversion [3, 6, 7]. Bluetongue virus causes significant economic losses in domestic ruminant production by both direct commodity loss and strict international livestock trade restrictions [3, 8, 9]. Countries free of BTV heavily monitor, restrict or ban importation of foreign ruminants, emphasizing the importance of BTV on international livestock trade [8, 10–12]. Despite the growing recognition of BTV as an emerging pathogen, there are limited control tools available for *Culicoides* [7, 12–14].

Source reduction, or larval habitat modification, is generally recommended as the primary strategy for controlling vector fly species, yet this has been shown to have a negligible influence on adult *Culicoides* populations and subsequent BTV infections [14–17]. In the USA, *Culicoides sonorensis* Wirth and Jones, the primary BTV vector, develops in organically enriched mud and is frequently found in association with dairy wastewater ponds. Controlled studies examining the effect of wastewater pond removal on *C. sonorensis* populations exist, yet the removal of an entire pond from a dairy farm had no effect on the local adult midge population [16]. Cultural control practices, like stabling, can be effective to protect animals from exophilic *Culicoides* species, such as *Culicoides imicola* Kieffer in Africa [18] but are not practical or possible for all livestock. Furthermore, the exo- or endophily of most *Culicoides* species, including *C. sonorensis*, has not been well described, and so stabling may not be an effective control technique in all cases.

Due to the lack of effective physical, cultural and biological control tactics for *Culicoides*, producers looking to reduce BTV transmission risk are left with only chemical controls. Current chemical control options are predominately pyrethroids, and few are labeled specifically for *Culicoides* [14]. Available products labeled for biting flies generally include pour-ons, sprays, ear tags and space sprays, all with synthetic pyrethroid active ingredients such as deltamethrin, $\alpha$-cypermethrin or permethrin [15, 19]. Products labeled for use on sheep may be especially ineffective against *Culicoides*, as pour-on and dip treatments spread poorly from the initial application site through thick wool fiber [1]. Similarly, topical repellents have been shown to be ineffective for use against biting midges in horses, as studies have found no reduction in biting rate, recurrent dermatitis or frequency of arbovirus transmission in treated equines [20–22]. To date, the most effective tool against *Culicoides* biting midges is cypermethrin- or deltamethrin-impregnated ear tags, which provide a toxic effect for up to 21 days in cattle and 10 days in sheep, respectively [23, 24]. The absence of alternatives to pyrethroids fosters the development of resistant biting midge populations through overexposure and lack of proper chemical rotation [25]. Adding another class of synthetic chemicals into treatment rotation is essential, and the novel isoxazoline, fluralaner, demonstrates potential in filling this need.

Isoxazoline insecticides, including fluralaner and afoxolaner, are ligand-gated chloride channel antagonists that disrupt nerve function. In the US, fluralaner is currently labeled for use against ectoparasites in companion animals under the trade name Bravecto^™^ (Merck Animal Health, Madison, NJ) and in Europe against poultry ectoparasites as Exzolt^™^ (Merck & Co., Rahway, NJ) [26–28]. Both Bravecto and Exzolt are systemic insecticides and are administered orally to animals. Ectoparasites are killed after ingesting the compound in a blood meal. However, laboratory studies have shown activity against off-label species such as house flies, mosquitoes and Triatomine kissing bugs [26, 28–33]. With its high efficacy against a range of both hematophagous and non-parasitic arthropods, and its novel mode of action, fluralaner presents a potential new tool for vector control. In this study we evaluated the susceptibility of adult *C. sonorensis* to topical, contact and oral applications of fluralaner to determine whether isoxazoline insecticides show promise for controlling biting midges on livestock.

## Methods

The insects used for this study were from a laboratory strain of *C. sonorensis* (Van Ryn), established from California in 1995. This strain has no known prior isoxazoline exposure. Midges were reared at 25 °C and a 12:12 light:dark cycle in a climate-controlled insectary and were provided 10% sucrose solution ab libitum as adults. At 1–3-days post emergence, non-blood-fed, nulliparous females were cold-anesthetized and counted prior to treatment. Preliminary exposure trials were performed to determine the appropriate range of concentrations to use for all susceptibility assays. Fluralaner dilutions were chosen predicated by the criteria that symptoms of acute toxicity (inability to fly, walk or stand) were observed at 1-h post-exposure for individuals treated with the highest dose in these preliminary experiments. One hundred milligrams of technical grade fluralaner (BOCSCI Inc., Shirley, NY) was formulated in either 1 ml HPLC-grade acetone (contact and topical trials) or DMSO (oral trials) and then serially diluted to the appropriate concentration. Concentration ranges used for contact, topical and oral susceptibility tests were 0.001–100 mg/ml, 0.001–10 mg/ml and 10–1000 ng/ml, respectively.

### Contact susceptibility

To assess contact toxicity of fluralaner, WHO cone assays were appropriately modified for *Culicoides* [22, 34, 35]. Fluralaner solution was diluted to 0.001, 0.01, 0.1, 1, 10 and 100 mg/ml; 100 µl of each dilution was applied to 26 × 31 mm Whatman no. 1 filter papers and allowed to completely dry for 5 min before being used in the bioassays. Control filter papers were treated with 100 µl acetone. Treated filter papers were placed in 50-mm disposable Petri dishes and covered with small polypropylene funnels (3.0 × 0.6 × 4 cm). Five adult *C. sonorensis* females were introduced to each cone, after which the neck of the funnel was plugged with cotton. Petri dishes and cones were placed flat on the laboratory bench. Midges were held in the cones for a contact exposure time of 6 min under ambient laboratory conditions. Midges from each replicate were then aspirated out of the cones into individual 120-ml cardboard containers with mesh lids and provided with 10% sucrose solution ab libitum. Cups were held in a 61.7 × 45.2 × 26.7-cm plastic box with a lid, with open containers of deionized water to increase relative humidity. The percent knockdown (%KD) in each replicate was recorded after 1 h, in which knocked-down individuals showed signs of acute toxicity (unable to walk, stand or fly). Mortality was assessed at 24 h, and dead individuals were considered moribund or unresponsive to stimuli. Four replicates per treatment were used, and contact toxicity bioassays were repeated six times.

### Topical susceptibility

To assess topical toxicity of fluralaner on midges, WHO topical toxicity assays were appropriately modified from the standard mosquito procedures [28, 35]. Fluralaner stock solutions were prepared in the same manner as in contact assays, and diluted to 0.001, 0.01, 0.1, 1 and 10 mg/ml. A Nanoject II automated injector (Drummond Scientific Co., Broomall, PA) was used to administer 20 nl of the fluralaner dilution to the lateral thorax of 20 adult females per treatment group with a pulled glass capillary needle. Control midges received 20 nl acetone. The 20-nl droplet volume was chosen because it is 1/5 of the standard volume used in mosquito toxicity trials [35], accounting for the relative size of *C. sonorensis*. After treatment, midges were transferred to 475-ml cardboard containers with mesh lids, provided with 10% sucrose solution ab libitum, and held in the same manner as in contact toxicity assays. Knockdown and mortality for each treatment group was assessed and recorded at 1 and 24 h, respectively. Twenty individuals per treatment group were used, and topical toxicity bioassays were repeated four times.

### Oral susceptibility

To assess oral toxicity, females were offered a blood meal spiked with fluralaner in an artificial membrane feeding system; 100 mg of technical grade fluralaner was dissolved in DMSO to the concentration of 2 mg/ml. This solution was further diluted in PBS (Sigma-Aldrich, St. Louis, MO) to 2000 ng/ml [36] to create a stock solution. This fluralaner spiked PBS stock solution was then diluted in defibrinated sheep blood (Carolina Biological Supply Co., Burlington, NC) to concentrations of 10, 30, 50, 70, 300, 400 or 1000 ng/ml. Blood meals were added to glass membrane feeders hooked up to a recirculating water bath set to 37 °C with a stretched Parafilm membrane. Concentration groups of ten 1–3-day-old females were allowed to feed for 30 min, using five replicates per concentration. After feeding, midges were anesthetized with CO_2_, and unengorged females were removed. Each replicate then included 1–8 engorged midges, which were held for 96 h and provided with 10% sucrose solution ab libitum, with mortality assessed at 1 h and every 24 h thereafter. Oral toxicity trials were repeated three times.

### Statistical analysis

Data were analyzed using R (version 4.1.3). A generalized linear model (GLM) was used to determine whether an interaction between trial and treatment was present prior to pooling data from multiple trials. An analysis of variance (ANOVA), followed by Tukey’s honestly significant difference test with a Bonferroni correction for multiple comparisons, was conducted to determine whether any exposure to any concentration of fluralaner significantly increased midge mortality compared to the control for each of the three exposure types and whether concentrations differed from each other. If fluralaner exposure significantly increased *C. sonorensis* mortality, probit analysis was used to predict the lethal concentration of fluralaner leading to 50% and 90% knockdown at 1 h (KD_50/90_) and 50% and 90% mortality at 24 h post-exposure (LC_50/90_) [37]. Model fit was assessed using a Chi-square test, and a heterogeneity factor was incorporated if the model failed the goodness of fit test (*P* > 0.05). Abbott’s correction was used to correct for control mortality if it exceeded 5% [38]. If control mortality exceeded 20%, the experiment was repeated. For all analyses, *P* < 0.05 was considered to be statistically significant.

## Results

### Contact susceptibility

**Fig. 1:**
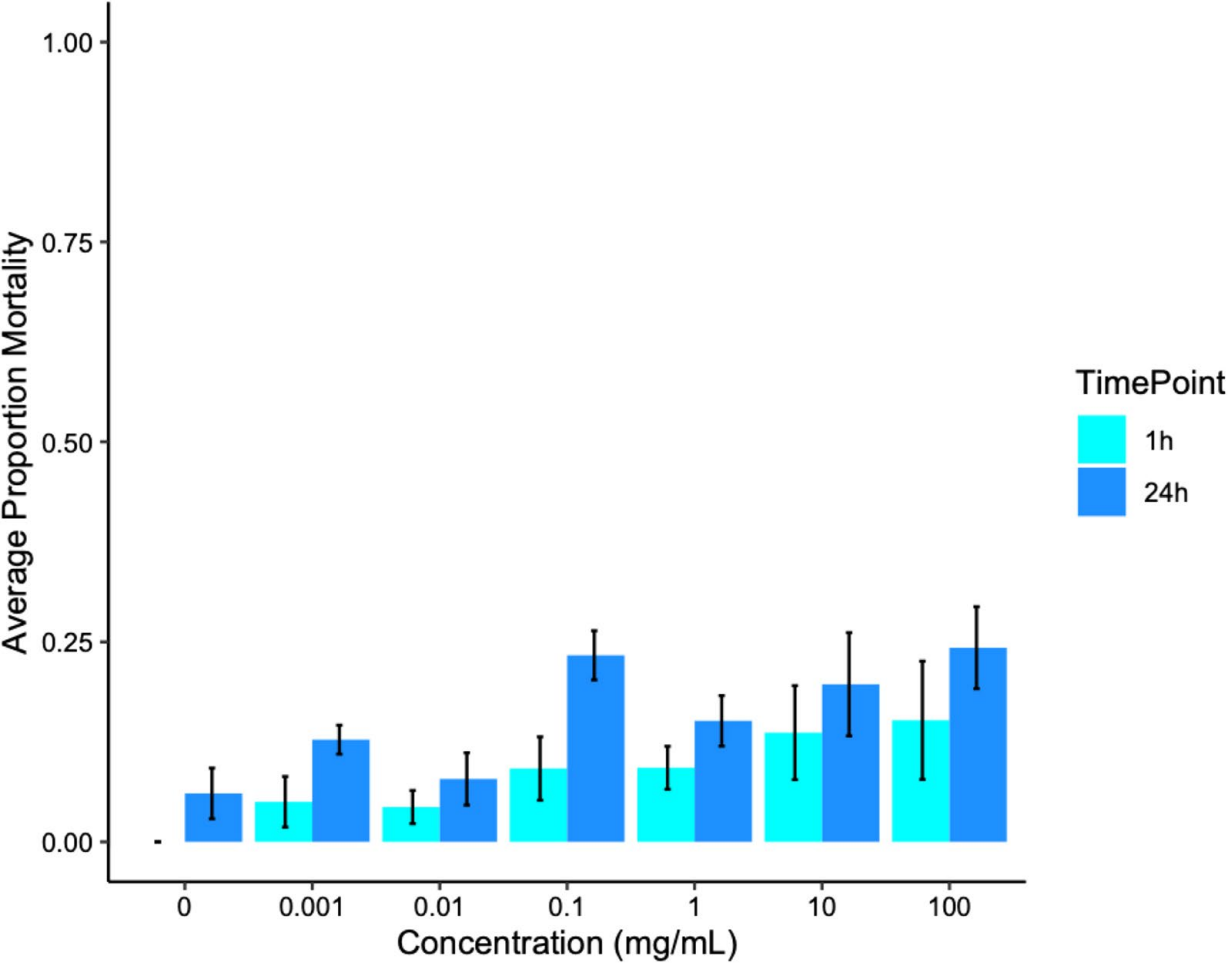
Average mortality after contact exposure to fluralaner at concentrations of 0–100 mg/ml for *Culicoides sonorensis* at 1 h (*n* = 777 total individuals tested) and 24 h (*n* = 773 total individuals tested); 1 h is represented by light blue and 24 h by dark blue. Error bars represent standard deviation and asterisks represent significant differences between control (concentration = 0 mg/ml) and treatment at each concentration (***P < 0.0005). Concentrations not marked with asterisks did not differ significantly between control and treatment

At 1 h post-exposure there was no significant difference in knockdown or mortality for any concentration group when compared to the control group (Fig. 1). Because contact exposure had no effect on *C. sonorensis* survival at 1 h, the LC_50_ could not be estimated. At 24 h post-exposure, there was still low *C. sonorensis* mortality in contact assays, with average mortality not exceeding 24.3%, even at the highest concentration tested (100 mg/ml). At 24 h, contact exposure to fluralaner did significantly increase *C. sonorensis* mortality compared to the control (*P* = 0.012, F (6,33) = 3.28). After correcting for multiple comparisons, no treatments significantly differed, therefore, the 24 h LC_50_ could not be estimated using the probit analysis.

### Topical susceptibility

**Fig. 2:**
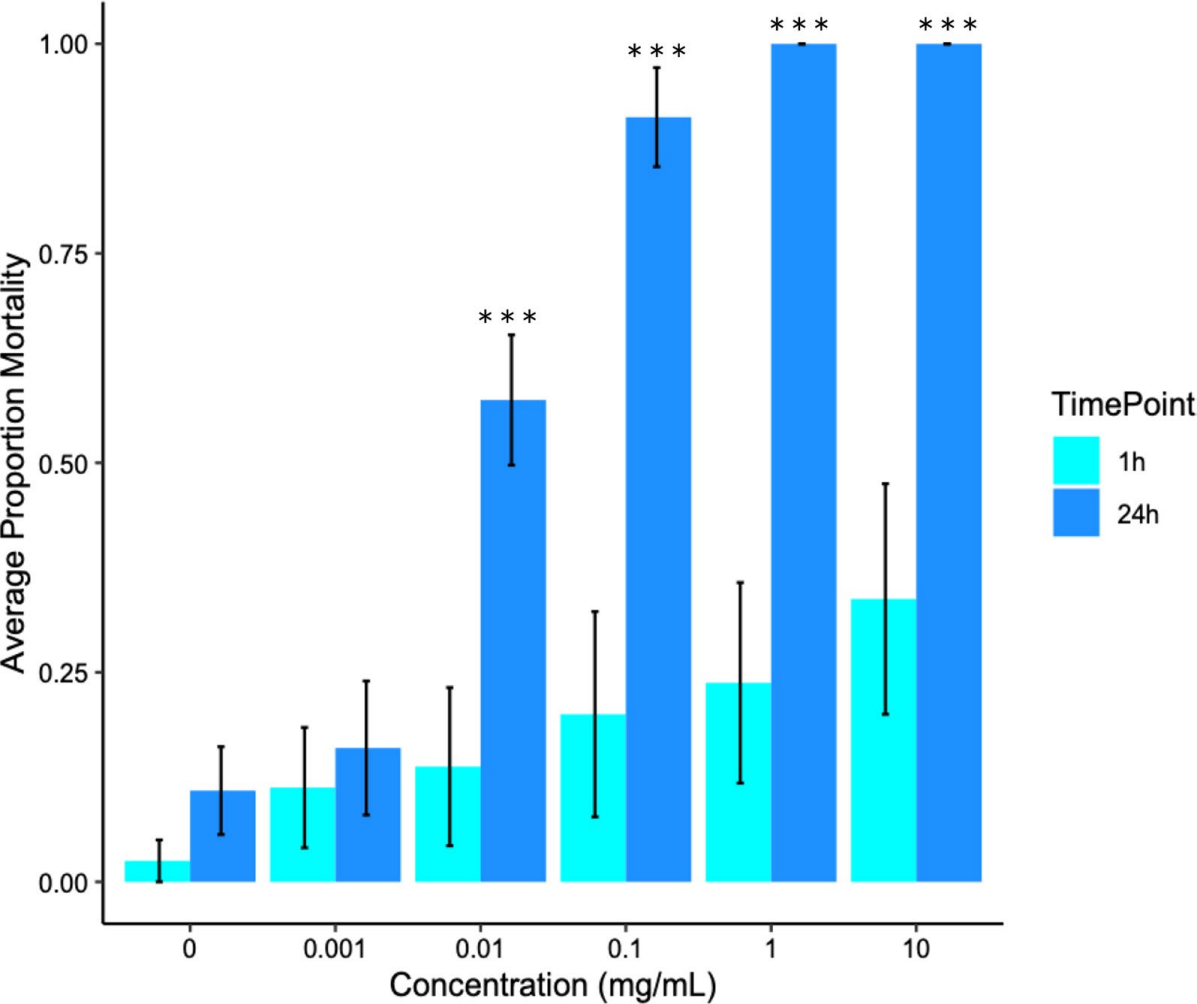
Average mortality after topical exposure to fluralaner at concentrations of 0–10 mg/ml for *Culicoides sonorensis* at 1 h (*n* = 480 total individuals tested) and 24 h (*n* = 475 total individuals tested); 1 h is represented by light blue and 24 h by dark blue. Error bars represent standard deviation, and asterisks represent significant differences between control (concentration = 0 mg/ml) and treatment at each concentration (***P < 0.0005). Concentrations not marked with asterisks did not differ significantly between control and treatment

At 1 h post-exposure there was no significant difference in knockdown or mortality for any treatment group when compared to the control group in topical application experiments (Fig. 2). Topical exposure to fluralaner did significantly increase *C. sonorensis* mortality compared to the control in a dose-dependent manner at 24 h. Concentrations of 0.01, 0.1, 1 and 10 mg/ml fluralaner had significantly higher mortality at 24 h (*P* < 0.0001, F (7,20) = 44.4) than controls (Fig. 2). One hundred percent mortality after topical exposure to concentrations of ≥ 1 mg/ml was observed at 24 h post-exposure. The estimated LC_50_ and LC_90_ (24 h) were 0.0108 mg/ml and 0.084 mg/ml, respectively (Table 1).

**Table 1:** Topical toxicity of fluralaner on *Culicoides sonorensis* at 24 h

Timepoint	*n*	Slope (SD)	LC_50_ (95% CI)	LC_90_ (95% CI)	*X*^2^ (df)	*P*-value
24 h	400	1.45 (0.15)	0.0108 (0.008, 0.015)	0.0842 (0.053, 0.158)	1.33 (3)	0.25

*n* is the total number of individuals exposed in topical toxicity bioassays. LC_50_ and LC_90_ were estimated using probit analysis. LC values are expressed as mg/ml

### Oral susceptibility

**Fig. 3:**
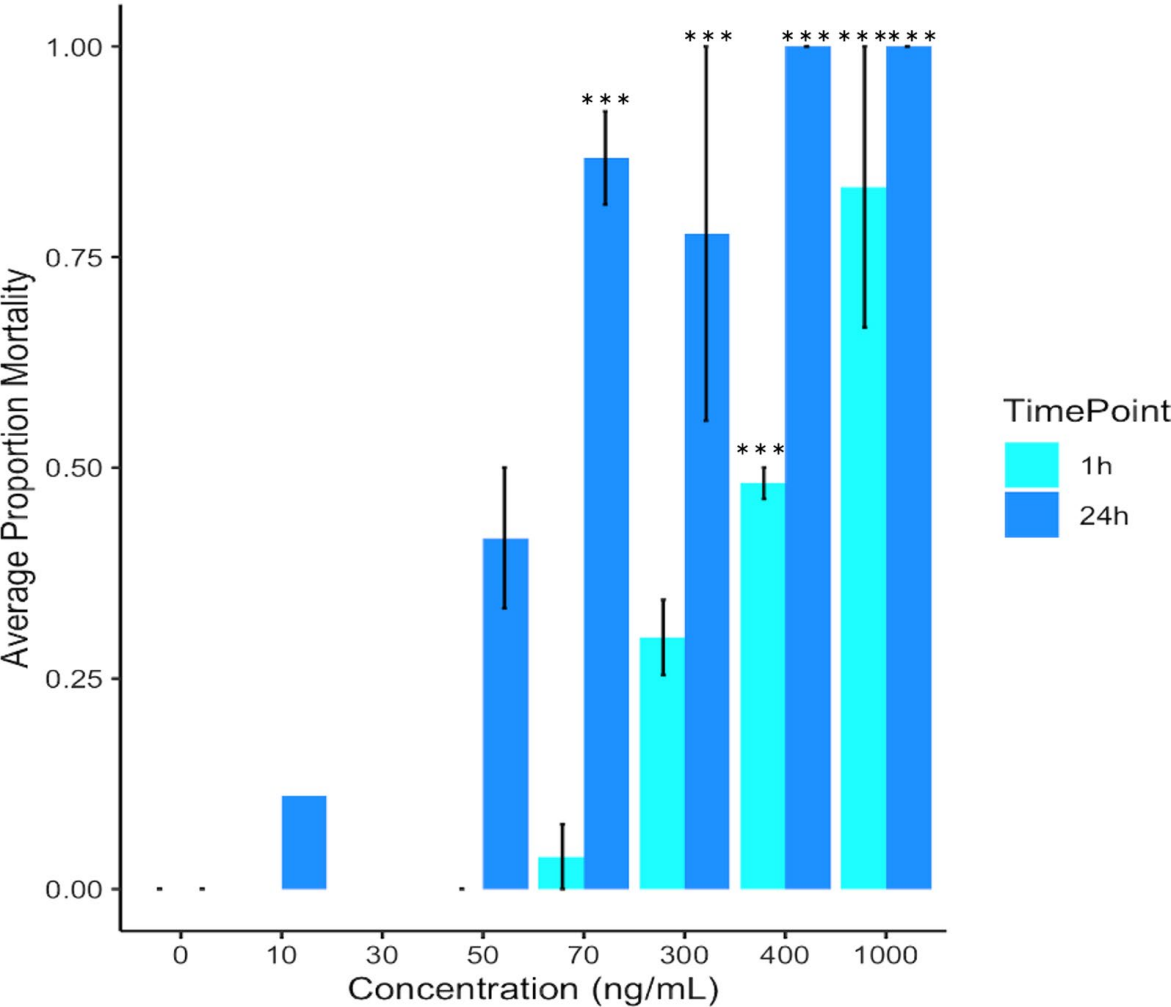
Average mortality after oral exposure to fluralaner at concentrations of 0–1000 ng/ml for *Culicoides sonorensis* at 1 h (*n* = 144 total individuals tested) and 24 h (*n* = 144 total individuals tested); 1 h is represented by light blue and 24 h by dark blue. Error bars represent standard deviation and asterisks represent significant differences between control (concentration = 0 ng/ml) and treatment at each concentration (***P < 0.0005). Concentrations not marked with asterisks did not differ significantly between control and treatment

Oral exposure to fluralaner in an artificial blood meal significantly increased *C. sonorensis* mortality compared to the control starting at 1 h post-exposure (*P* < 0.0001, F (7, 56) = 14.6) at concentrations of 400 and 1000 ng/ml (Fig. 3), although lower concentrations did not differ from the control. Mortality increased in a dose-dependent manner, with a predicted 1 h LC_50_ of 441.3 ng/ml and LC_90_ of 1371.07 ng/ml (Table 2). At 24 h post-exposure, exposures to 70, 300, 400 and 1000 ng/ml significantly increased mortality compared to the control (*P* < 0.0001, F (7, 56) = 81.9). For oral exposure, the predicted 24 h LC_50_ and LC_90_ were 14.42 ng/ml and 60.65 ng/ml, respectively (Table 2). At time points beyond 24 h, no additional mortality was recorded in any group, with the exception of one individual in the control group that died at 96 h. No notable difference in feeding rate was observed between the control groups and treatment groups.

**Table 2:** Oral toxicity of fluralaner on *Culicoides sonorensis* at 1 h and 24 h

Timepoint	*n*	Slope (SD)	LC_50_ (95% CI)	LC_90_ (95% CI)	*X*^2^ (df)	*P*-value
1 h	106	2.06 (0.56)	441.3 (343.5, 624.1)	1371.1 (870.2, 3866.8)	1.02 (2)	0.60
24 h	76	2.05 (0.63)	14.4 (2.50, 26.4)	60.7 (40.0, 133.6)	0.008 (1)	0.93

* n* is the total number of individuals exposed in oral toxicity bioassays. LC_50_ and LC_90_ were estimated using probit analysis. LC values are expressed as ng/ml

## Discussion

Producers are encouraged to rotate products from different chemical classes as part of an integrated pest management (IPM) program, as repeated exposure to compounds with the same mode of action unintentionally selects for mutations that allow for survival, producing resistant populations [25, 39]. Insecticide resistance is increasingly problematic for producers, especially those with as few available control tools as ruminant producers [40–42]. Chemical insecticides are often the primary choice for producers seeking to control *Culicoides*, yet only a handful of active ingredients are available to target biting midges, preventing the use of an effective rotation program. Furthermore, pyrethroid insecticides have shown limited efficacy at controlling *Culicoides* in the field [1, 14, 20, 21] or preventing BTV transmission in endemic areas [19]. New products with additional modes of action are needed for *Culicoides* control on ruminant livestock. Isoxazolines are noncompetitive antagonists and potent inhibitors of γ-aminobutyric acid (GABA) and glutamate-gated chloride channels, leading to paralysis and death through prolonged hyperpolarization [26, 43, 44]. The isoxazoline mode of action is vastly distinct from other common insecticides, such as pyrethroids, which bind voltage-gated sodium channels, or carbamates, organophosphates and neonicotinoids, which all inhibit the hydrolytic enzyme, acetocholinesterase [39, 43, 45]. Here, we characterized the susceptibility of *C. sonorensis* to the isoxozaline insecticide fluralaner using three insecticide susceptibility bioassays.

Susceptibility assays were chosen to reflect potential application methods to be used in a field setting. Contact toxicity was assessed by using a modified WHO cone assay, which simulates exposure through landing on an insecticide treated surface. This protocol for assessment was first used to evaluate efficacy of insecticide-treated bednets against *Anopheles* mosquitoes [35] but was adapted for the smaller size of *Culicoides* in our study and interpreted as representative of midges landing on an animal treated with an insecticidal pour-on or dip product. *Culicoides sonorensis* mortality in the contact susceptibility experiments was the lowest and most variable of the three bioassays. Contact exposure to fluralaner did not significantly increase *C. sonorensis* mortality, and even at the highest concentration tested (100 mg/ml) we only observed an average of 24.3% mortality at 24 h (Fig. 1). Likewise, fluralaner (24 h LD_50_: 13 ng/cm^2^) underperformed compared with fipronil (24 h LD_50_: 10 ng/cm^2^) in WHO contact toxicity bioassays with *Aedes aegypti* L. (Diptera: Culicidae) mosquitoes, demonstrating low cuticular absorption [28, 46].

Cuticular absorption because of contact exposure is inherently variable, both in vitro and in vivo [47]. Tarsal contact on the treated surface is highly variable depending upon time of direct contact, such that the cones can serve as an untreated resting place for exposed insects [47]. Previous contact assays conducted in our laboratory using permethrin have yielded more uniform levels of mortality than observed here with fluralaner (EGM, personal observation), suggesting that the variation observed in this study is at least in part due to fluralaner itself, as its large molecular size (556.3 g/mol) and high lipophilicity (log *p* = 5.0) contribute to limited cuticular penetration, as demonstrated in studies on mosquitoes and house flies [28, 46]. Because cuticular absorption of fluralaner is so low, the limited exposure from contact applications is likely to be insufficient in producing adequate adult mortality.

Based on our results, the contact LC_50/90_ of fluralaner for *C. sonorensis* at 24 h would be exceptionally high, beyond what was tested here. The concentration required would be neither practical nor feasible, as it would far exceed the saturation point in polar and organic solvents (DMSO: 100 mg/ml, ethanol: 25 mg/ml) [48]. Fluralaner in this amount would be hazardous for applicators as well as exceedingly likely to contaminate the surrounding environment [49–51]. Lastly, when considering a product’s potential for commercialization, this method of exposure for fluralaner is impractical. We utilized a 6-min exposure time in our experiments. Accurate measures of *Culicoides* feeding time to repletion are unavailable in the literature, but an uninterrupted contact time of > 6 min during blood-feeding is unlikely. In colony, we have observed that our *C. sonorensis* feed to repletion in ≤ 3 min. While this time is likely shorter than the duration of midge feeding under field conditions, most telmophagous insects feed quickly to reduce the potentially dangerous time they spend on a host [52, 53].

Fluralaner topical toxicity was also assessed for adult *C. sonorensis*. Ultra-low volume (ULV) sprays are routinely used for control of adult flying insects such as mosquitoes [54–56]. With this application method, insects are directly contacted by droplets of insecticide rather than being exposed to residuals. We assessed *C. sonorensis* topical susceptibility by directly applying a 20 nl drop of diluted fluralaner to the thorax to simulate direct exposure through nanodroplet contact; 100% mortality was consistently achieved at concentrations of 1 mg/ml at 24 h (Fig. 2). The LC_50_ for this method at 24 h was estimated to be 0.0108 mg/ml, indicating that topical exposure to fluralaner is a more practical application method for *Culicoides* control than surface contact with residuals (Table 1). A number of studies have assessed topical toxicity of fluralaner in other off-label pestiferous fly species, and many have shown limitations due to cuticular penetration [28, 46]. Similar to contact exposures, fipronil outperformed fluralaner after topical application to the thorax of *Ae. aegypti*, with the 24 h LD_50_ of each being 0.062 ng/mg and 1.3 ng/mg, respectively [28]. Fluralaner’s topical toxicity in adult stable flies (Diptera: Muscidae: *Stomoxys calcitrans*), horn flies (Diptera: Muscidae: *Haematobia irritans*) and house flies (Diptera: Muscidae: *Musca domestica*) has also been assessed [26]. The 24 h fluralaner LD_50_ for stable flies was 34.5 ng/fly while the permethrin LD_50_ was 0.76 ng/fly. By contrast, fluralaner (24 h LD_50_ 4.62 ng/fly) outperformed permethrin (24 h LD_50_ 10.4 ng/fly) in horn flies. House fly susceptibility varied by strain, with some evidence of cross-resistance in permethrin-resistant flies, though permethrin-susceptible flies had equivalent permethrin and fluralaner LD_50_ concentrations (~ 17.0 ng/fly). Fluralaner susceptibility is also lower in dieldrin-resistant (24 h LD_50_ 1.01 ng/mg) than susceptible house flies (24 h LD_50_ 0.85 ng/mg) [43].

As opposed to contact trials, behaviors indicating acute toxicity were immediately observed after topical exposure. For topical toxicity assays, concentrations of 0.1 mg/ml led to the display of acute toxicity in adult female *C. sonorensis* at just 1 h post-exposure. Symptoms of acute toxicity in midges include the inability to fly, inability to walk, twitchy wings or legs, and the inability to stand. An adulticide product formulated to these concentrations is more effective as demonstrated by the bioassays, but environmental contamination is of critical concern for this application type. To date, fluralaner is classified as “persistent/very persistent in soil and aerobic freshwater sediments,” indicating significant concern for ground water and soil contamination if applied using this method [50, 57]. Notably, droplet size is a limitation of the assay used here as 20 nl is on the larger end of ULV sprayer droplet size and is not the ideal size for effective control [55, 56]. Additional studies could assess susceptibility to fluralaner in this manner using ULV cage assays to develop more accurate LC_50/90_ estimates for topical toxicity. The residual effects of fluralaner on wool have yet to be evaluated following topical application, but future studies could address this. Midge feeding rates after contact and topical exposure were not assessed in this study. Topical applications of permethrin on cattle have been shown to have no effect on *Culicoides* engorgement rates [57]. The effects of cuticular absorption of fluralaner on midge feeding are currently unknown. One potential limitation of this study is that only one laboratory population of *C. sonorensis* was used. Although it is unlikely that any field population of *C. sonorensis* has developed resistance to fluralaner because of its current limitation to pets in the US, there may be variation in the natural level of susceptibility among populations [58].

In its currently labeled formulations, fluralaner is approved for use as a systemic insecticide and acaricide in companion animals and poultry. The active ingredient is metabolized by the animal into the blood stream, and ectoparasites are exposed when they feed on the treated animal. Due to the concerns about environmental contamination with fluralaner, systemic treatment of livestock is likely to be the most practical application route for *Culicoides* control, and a number of studies have pursued interest in this exposure route for other insect vectors. Following a fluralaner-spiked blood meal given to mosquitoes, the LC_50_ values (*Anopheles stephensi* LC_50_: 100 ng/ml; *An. albimanus* LC_50_: 32 ng/ml) displayed comparable efficacy to the predominately used phenylpyrazole, fipronil (*An. stephensi* LC_50_: 123 ng/ml; *An. albimanus* LC_50_: 23 ng/ml) [36]. Conversely, fipronil outperformed fluralaner 100-fold when *Ae. aegypti* were provided insecticide-spiked sucrose [28]. The oral LD_50_ of fluralaner in *M. domestica* strains with varying resistance to permethrin ranged from 1.47 to 6.93 μg/g sugar, exceeding imidacloprid oral toxicity 9- to 118-fold [26]. We found that ingestion of fluralaner in an artificial blood meal resulted in the highest mortality and lowest LC values. Beginning at just 1 h post-exposure, concentrations of  ≥ 400 ng/ml significantly increased *C. sonorensis* mortality compared to controls. The 24 h LC_50_ for orally ingested fluralaner is 14.42 ng/ml, which is considerably lower than our estimated 24 h topical LC_50_ value (0.0108 mg/ml). This shows that an exposure route that bypasses the cuticle is notably more toxic and therefore effective for control of *Culicoides* biting midges.

At present, mammalian pharmacokinetic studies following ingestion of fluralaner are limited and have only been conducted in dogs and black bears [59, 60]. However, our calculated LC_50_ for *C. sonorensis* at 1 h and 24 h post-ingestion falls well within the known maximum plasma concentrations for fluralaner for these species (dogs: 3948 ng/ml, black bears: 14550 ng/ml), suggesting that midges feeding on fluralaner-treated animals would consume a fatal dose. When considering fluralaner’s potential as a livestock insecticide, other factors beyond the laboratory susceptibility of hematophagous arthropods should be considered, including mean residence time (MRT). MRT is the average time a drug molecule spends in circulation in a vertebrate. MRT is used to calculate acceptable levels of a compound in tissue to be consumed in meat animals, in conjunction with re-application intervals. Fluralaner’s pharmacokinetic profile is characterized by a long systemic persistence, with the MRT of orally administered fluralaner being 15 days in dogs and 7 days in black bears [59, 60]. However, as we propose use in livestock animals, MRT and the frequency of application needed for successful control of *Culicoides* need to be studied. As withdrawal period requirements for meat animals present unique yet definitive challenges, pharmacokinetic studies of isoxazoline drugs need to be pursued and finalized for livestock before fluralaner can be used as a management strategy in this manner. Conversely, livestock not raised for consumption, such as hair sheep, would directly benefit from administering oral fluralaner as a component of a BTV disease management program.

## Conclusion

*Culicoides sonorensis* shows high susceptibility to orally ingested fluralaner, moderate susceptibility to topical applications of fluralaner and low susceptibility to residual contact with fluralaner. Systemic livestock products using fluralaner or other isoxazoline insecticides may be an effective option for controlling *Culicoides* and preventing BTV transmission. Future studies should focus on the pharmacokinetics of fluralaner in meat animals to determine its practical use for animal agriculture. Additionally, work assessing the residual toxicity of fluralaner metabolites in animal excrement on nontarget, coprophagous insects prior to any product implementation would be recommended, as this may inadvertently lead to the development of isoxazoline-resistant pests (e.g. *Stomoxys calcitrans*). Yet, despite the research that remains to be done, fluralaner shows promise as a novel control tool for ruminant livestock producers attempting to control for biting midge populations.

## Data Availability

Data supporting these conclusions are included within the article.

## Abbreviations

BTV: Bluetongue virus
EHDV: Epizootic hemorrhagic disease virus
LC: Lethal concentration
KD: Knockdown
PBS: Phosphate-buffered saline
DMSO: Dimethyl sulfoxide
ULV: Ultralow volume spray
IPM: Integrated pest management
MRT: Mean residence time

## References

[1] Carpenter S, Groschup MH, Garros C, Felippe-Bauer ML, Purse BV (2013). *Culicoides* biting midges, arboviruses and public health in Europe. Antivir Res.

[2] Maclachlan NJ (2011). Bluetongue: history, global epidemiology, and pathogenesis. Prev Vet Med.

[3] Mellor PS, Boorman J, Baylis M (2000). *Culicoides* biting midges: their role as arbovirus vectors. Annu Rev Entomol.

[4] Alkhamis MA, Aguilar-Vega C, Fountain-Jones NM, Lin K, Perez AM, Sánchez-Vizcaíno JM (2020). Global emergence and evolutionary dynamics of bluetongue virus. Sci Rep.

[5] Mills MK, Ruder MG, Nayduch D, Michel K, Drolet BS (2017). Dynamics of epizootic hemorrhagic disease virus infection within the vector, *Culicoides*
*sonorensis* (Diptera: Ceratopogonidae). PLoS ONE.

[6] Stevens G, McCluskey B, King A, O’Hearn E, Mayr G (2015). Review of the 2012 epizootic hemorrhagic disease outbreak in domestic ruminants in the United States. PLoS ONE.

[7] Sick F, Beer M, Kampen H, Wernike K (2019). *Culicoides* biting midges—underestimated vectors for arboviruses of public health and veterinary importance. J Viruses.

[8] MacLachlan NJ, Jagels G, Rossitto PV, Moore PF, Heidner HW (1990). The pathogenesis of experimental bluetongue virus infection of calves. Vet Pathol.

[9] Mullens BA, Gerry AC, Lysyk TJ, Schmidtmann ED (2004). Environmental effects on vector competence and virogenesis of bluetongue virus in *Culicoides*: interpreting laboratory data in a field context. Vet Ital.

[10] Giovannini A, Conte A, Calistri P, Di Francesco CA, Caporale V (2004). Risk analysis on the introduction into free territories of vaccinated animals from restricted zones. Vet Ital.

[11] MacLachlan NJ, Osburn BI (2006). Impact of bluetongue virus infection on the international movement and trade of ruminants. J Am Vet Med Assoc.

[12] Papadopoulas O, Mellor PS, Mertens PP, Mellor P, Baylis M, Mertens P (2009). Bluetongue control strategies. Bluetongue.

[13] Maclachlan NJ, Mayo CE (2013). Potential strategies for control of bluetongue, a globally emerging, *Culicoides*-transmitted viral disease of ruminant livestock and wildlife. Antivir Res.

[14] Purse BV, Carpenter S, Venter GJ, Bellis G, Mullens BA (2015). Bionomics of temperate and tropical *Culicoides* midges: knowledge gaps and consequences for transmission of *Culicoides*-borne viruses. Annu Rev Entomol.

[15] Carpenter S, Mellor PS, Torr SJ (2008). Control techniques for *Culicoides* biting midges and their application in the UK and northwestern Palaearctic. Med Vet Entomol.

[16] Mayo CE, Osborne CJ, Mullens BA, Gerry AC, Gardner IA, Reisen WK, Barker CM, MacLachlan NJ (2014). Seasonal variation and impact of waste-water lagoons as larval habitat on the population dynamics of *Culicoides*
*sonorensis* (Diptera: Ceratpogonidae) at two dairy farms in northern California. PLoS ONE.

[17] Mullens BA, Rodriguez JL (1989). Response of *Culicoides*
*variipennis* (Diptera: Ceratopogonidae) to water level fluctuations in experimental dairy wastewater ponds. J Med Entomol.

[18] Meiswinkel R, Baylis M, Labuschagne K (2000). Stabling and the protection of horses from *Culicoides*
*bolitinos* (Diptera: Ceratopogonidae), a recently identified vector of African horse sickness. Bull Entomol Res.

[19] Mullens BA, Gerry AC, Velten RK (2001). Failure of a permethrin treatment regime to protect cattle against bluetongue virus. J Med Entomol.

[20] De Raat IJ, Van Den Boom R, Van Poppel M, van Oldruitenborgh-Oosterbaan MM (2008). The effect of a topical insecticide containing permethrin on the number of *Culicoides* midges caught near horses with and without insect bite hypersensitivity in the Netherlands. Tijdschr Diergeneeskd.

[21] Lincoln VJ, Page PC, Kopp C, Mathis A, Von Niederhäusern R, Burger D, Herholz C (2015). Protection of horses against *Culicoides* biting midges in different housing systems in Switzerland. Vet Parasitol.

[22] Page PC, Labuschagne K, Nurton JP, Venter GJ, Guthrie AJ (2009). Duration of repellency of N, N-diethyl-3-methylbenzamide, citronella oil and cypermethrin against *Culicoides* species when applied to polyester mesh. Vet Parasitol.

[23] Liebisch G, Liebisch A (2008). Efficacy of Flectron-eartags (cypermethrin) for control of midges (*Culicoides*) as the vectors of bluetongue virus in cattle: field studies and biossays. Deutsche Tierarztliche Wochenschrift.

[24] Venail R, Mathieu B, Setier-Rio ML, Borba C, Alexandre M, Viudes G, Garros C, Allene X, Carpenter S, Baldet T, Balenghien T (2011). Laboratory and field-based tests of deltamethrin insecticides against adult *Culicoides* biting midges. J Med Entomol.

[25] Barzman M, Bàrberi P, Birch AN, Boonekamp P, Dachbrodt-Saaydeh S, Graf B, Hommel B, Jensen JE, Kiss J, Kudsk P, Lamichhane JR (2015). Eight principles of integrated pest management. Agron Sustain Dev.

[26] Burgess ER, Geden CJ, Lohmeyer KH, King BH, Machtinger ET, Scott JG (2020). Toxicity of fluralaner, a companion animal insecticide, relative to industry-leading agricultural insecticides against resistant and susceptible strains of filth flies. Sci Rep.

[27] Casida JE, Durkin KA (2015). Novel GABA receptor pesticide targets. Pestic Biochem Phys.

[28] Jiang S, Tsikolia M, Bernier UR, Bloomquist JR (2017). Mosquitocidal activity and mode of action of the isoxazoline fluralaner. Int J Environ Res Pub Health.

[29] Gassel M, Wolf C, Noack S, Williams H, Ilg T (2014). The novel isoxazoline ectoparasiticide fluralaner: selective inhibition of arthropod γ-aminobutyric acid-and L-glutamate-gated chloride channels and insecticidal/acaricidal activity. Insect Biochem Mol Biol.

[30] Laiño MA, Cardinal MV, Enriquez GF, Alvedro A, Gaspe MS, Gürtler RE (2019). An oral dose of Fluralaner administered to dogs kills pyrethroid-resistant and susceptible Chagas disease vectors for at least four months. Vet Parasitol.

[31] Miglianico M, Eldering M, Slater H, Ferguson N, Ambrose P, Lees RS, Koolen KM, Pruzinova K, Jancarova M, Volf P, Koenraadt CJ (2018). Repurposing isoxazoline veterinary drugs for control of vector-borne human diseases. PNAS.

[32] Taenzler J, de Vos C, Roepke RK, Frénais R, Heckeroth AR (2017). Efficacy of fluralaner against *Otodectes*
*cynotis* infestations in dogs and cats. Parasit Vectors.

[33] Weber T, Selzer PM, Meng CQ, Sluder AE (2018). Isoxazolines: a novel chemotype highly effective on ectoparasites. Ectoparasites: drug discovery against moving targets.

[34] Del Rio R, Venail R, Calvete C, Barcelo C, Baldet T, Lucientes J, Miranda MA (2014). Sensitivity of *Culicoides*
*obsoletus* (Meigen) (Diptera: Ceratopogonidae) to deltamethrin determined by an adapted WHO standard susceptibility test. Parasitol.

[35] World Health Organization (WHO). Guidelines for testing mosquito adulticides for indoor residual spraying and treatment of mosquito nets. 2006. http://apps.who.int/iris/handle/10665/69296. Accessed 10 May 2023.

[36] Dreyer SM, Vaughan JA (2022). Survival and fecundity of *Anopheles stephensi* and *Anopheles albimanus* mosquitoes (Diptera: Culicidae) after ingesting bovine blood containing various veterinary systemic parasiticides. J Med Entomol.

[37] Burgess ER, King BH, Geden CJ (2020). Oral and topical insecticide response bioassays and associated statistical analyses used commonly in veterinary and medical entomology. J Insect Sci.

[38] Abbott WS (1925). A method of computing the effectiveness of an insecticide. J Econ Entomol.

[39] Das SK (2013). Mode of action of pesticides and the novel trends–a critical review. Int J Agric Sci.

[40] Durel L, Estrada-Peña A, Franc M, Mehlhorn H, Bouyer J (2015). Integrated fly management in European ruminant operations from the perspective of directive 2009/128/EC on sustainable use of pesticides. Parasitol Res.

[41] Elzen GW, Hardee DD (2003). United States department of agriculture-agricultural research service research on managing insect resistance to insecticides. Pest Manag Sci.

[42] Harrup LE, Miranda MA, Carpenter S (2011). Advances in control techniques for *Culicoides* and future prospects. Vet Ital.

[43] Ozoe Y, Asahi M, Ozoe F, Nakahira K, Mita T (2010). The antiparasitic isoxazoline A1443 is a potent blocker of insect ligand-gated chloride channels. Biochem Biophys Res Comm.

[44] Zhao C, Casida JE (2014). Insect γ-aminobutyric acid receptors and isoxazoline insecticides: toxicological profiles relative to the binding sites of [3H] fluralaner, [3H]-4′-ethynyl-4-n-propylbicycloorthobenzoate, and [3H] avermectin. J Agric Food Chem.

[45] Stenersen J (2004). Chemical pesticides mode of action and toxicology.

[46] Norris RH, Baker OS, Burgess ER, Tarone A, Gerry A, Fryxell RT, Hinkle NC, Olds C, Boxler D, Wise KL, Machtinger ET (2023). Selection for, and characterization of, fluralaner resistance in the house fly. Musca domestica. Pestic Biochem Phys.

[47] Althoff RA, Huijben S (2022). Comparison of the variability in mortality data generated by CDC bottle bioassay, WHO tube test, and topical application bioassay using *Aedes*
*aegypti* mosquitoes. Parasit Vectors.

[48] Selleckchem: Fluralaner safety data sheet. 2013. https://www.selleckchem.com/datasheet/fluralaner-S647001-DataSheet.html. Accessed 03 April 2023.

[49] Diepens NJ, Belgers D, Buijse L, Roessink I (2023). Pet dogs transfer veterinary medicines to the environment. Sci Total Environ.

[50] Gastaldi MS, Felsztyna I, Miguel V, Sánchez-Borzone ME, García DA (2023). Theoretical and experimental study of molecular interactions of fluralaner with lipid membranes. J Agric Food Chem.

[51] Jia ZQ, Liu D, Sheng CW, Casida JE, Wang C, Song PP, Chen YM, Han ZJ, Zhao CQ (2018). Acute toxicity, bioconcentration, elimination and antioxidant effects of fluralaner in zebrafish. Danio rerio Environ Pollut.

[52] Beaty BJ, Marquardt WC (1996). The biology of disease vectors.

[53] Quate LW (1964). Phlebotomus sandflies of the Paloich area in the Sudan (Diptera, Psychodidae). J Med Entomol.

[54] Bonds JA (2012). Ultra-low-volume space sprays in mosquito control: a critical review. Med Vet Entomol.

[55] Mount GA, Biery TL, Haile DG (1996). A review of ultralow-volume aerial sprays of insecticide for mosquito control. J Am Mosq Control Assoc.

[56] Mount GA (1998). A critical review of ultralow-volume aerosols of insecticide applied with vehicle-mounted generators for adult mosquito control. J Am Mosq Control Assoc.

[57] Mullens BA, Velten RK, Gerry AC, Braverman Y, Endris RG (2000). Effects of permethrin and pirimiphos-methyl applied to cattle on feeding and survival of *Culicoides*
*sonorensis* (Diptera: Ceratopogonidae). Med Vet Entomol.

[58] Venail R, Lhoir J, Fall M, Del Río R, Talavera S, Labuschagne K, Miranda M, Pagès N, Venter G, Rakotoarivony I, Allène X (2015). How do species, population and active ingredient influence insecticide susceptibility in *Culicoides* biting midges (Diptera: Ceratopogonidae) of veterinary importance?. Parasit Vectors.

[59] Kilp S, Ramirez D, Allan MJ, Roepke RK, Nuernberger MC (2014). Pharmacokinetics of fluralaner in dogs following a single oral or intravenous administration. Parasit Vectors.

[60] Van Wick P, Papich MG, Hashem B, Dominguez-Villegas E (2020). Pharmacokinetics of a single dose of fluralaner administered orally to American black bears (*Ursus*
*americanus*). J Zoo Wild Med.

